# Differences in Physical Activity and Self-Rated Health Levels Based on Satisfaction with Physical Environment of Local Communities: From School to Lifelong Physical Education

**DOI:** 10.3390/healthcare12222244

**Published:** 2024-11-11

**Authors:** Byung-Kweon Chang, Se-Won Park, Seung-Man Lee

**Affiliations:** 1 Department of Physical Education, Seowon University, Cheongju 28674, Republic of Korea; jangbk82@gmail.com; 2 Department of Elementary Education, Korea National University of Education, Cheongju 28173, Republic of Korea; 3 Department of Sports Science, Hankyong National University, Anseong 17579, Republic of Korea

**Keywords:** KCHS, local community, physical environment, physical activity, self-rated health level

## Abstract

Background: In Korea, substantial disparities exist in physical environments across regions, exacerbating the polarization between metropolitan and provincial areas and urban and rural regions. Objectives: This study examines the differences in the physical activity and self-rated health of local communities in relation to satisfaction with the physical environment. By identifying and addressing these underlying causes of health disparities, the study provides foundational data to inform policy efforts. Methods: The study utilized data from the 2023 Community Health Survey conducted by the Korea Disease Control and Prevention Agency. It included 231,752 individuals aged 19 and older (105,754 men and 125,998 women) selected through a two-stage probability proportional systematic sampling method. One-on-one online interviews were conducted from 16 May to 31 July 2023. The physical environment was assessed based on public satisfaction with safety, natural and living environments, public transportation, and medical services. Study variables included subjective health status and physical activity, with subvariables for physical activity encompassing the duration of vigorous and moderate physical activity, walking, and flexibility exercises. For data analysis, MANOVA and ANOVA were conducted, with Bonferroni correction for multiple comparisons. Results: Considerable variations in physical activity were observed based on satisfaction with the local community environment. Regarding self-rated health, significant differences were found in safety levels, living environment, and medical services. Satisfaction with the physical environment positively influenced physical activity and self-rated health. Conclusion: Therefore, it is imperative to reduce disparities in the physical environment between regions and enhance residents’ satisfaction.

## 1. Introduction

In South Korea, disparities in physical environments exist across regions—between metropolitan areas and provinces and urban and rural regions—triggering conflicts among community members. This gap has widened recently, emerging as a significant social issue alongside generational and class polarization. Social costs are also increasing as a result of the deepening polarization and the resulting conflicts. The phenomenon is not limited to South Korea; similar patterns have been observed in many countries worldwide [1,2,3].

The physical environment of a community is shaped by factors such as community relations, safety, natural surroundings, living conditions, public transportation, and medical services. Disparities in the physical environment have wide-ranging impacts on the lives of community members. Collado [4] argued that easy access to medical services in the Philippines significantly impacts health and should be addressed through government policy. A case study of the Zaltbommel region by Bijloo [5] highlighted that community physical environments, such as community centers, contribute to individual rights and wellbeing in the Netherlands.

Health-related disparities, in particular, represent a critical issue requiring urgent attention. The concentration of fitness centers and medical services in specific areas has been identified as a contributing factor to social conflict. Therefore, this study explores the differences in physical activity and self-rated health (SRH)—key determinants of community health—to assess the significance of the physical environment in local communities.

Physical activity is an important aspect that has long been discussed in the fields of health science, public health, physical education, and pedagogy. Various studies have been conducted on the positive effects of physical activity and directions for promoting it. Global organizations, such as the International Society for Physical Activity and Health, have proposed eight investment areas for promoting physical activity, including whole-of-school programs, active transport, active urban design, healthcare, public education, sport and recreation, workplaces, and community-wide programs [6]. Additionally, studies have focused on the physical activity of marginalized or low-income community members [7,8]. Accordingly, large-scale empirical research should be conducted to identify specific physical environments that promote physical activity.

SRH is a subjective indicator where individuals assess their health status. It has been widely used in various studies owing to its simplicity, reliability, and validity. SRH plays a crucial role in predicting long-term health outcomes by providing a comprehensive evaluation of physical and mental health [9,10]. Previous studies have determined that physical environments in communities, such as green spaces and accessibility to public facilities, have a positive impact on individuals’ SRH [11,12]. However, many of these studies have limitations in that they focused solely on specific educational levels or age groups or involved a limited number of cases, making it difficult to generalize the results [13,14]. To overcome these limitations and maximize the advantages of SRH, it is imperative to conduct large-scale studies that include diverse population groups.

This study explored the differences in physical activity and self-rated health levels according to satisfaction with the physical environment of the local community. The study also aims to use large-scale regional and national data to monitor policy changes and foster environments that support increased physical activity [15,16]. In summary, the study seeks to explore the differences in physical activity and SRH according to satisfaction with the physical environment in local communities, provide fundamental data for policy efforts to identify and address regional health disparities, and clarify the conditions necessary for the expansion of physical education from school-based programs to lifelong physical education. For this purpose, the following research questions were formulated.

1. What are the differences in physical activity based on satisfaction with the physical environment of the local community?
2. What are the differences in self-rated health levels based on satisfaction with the physical environment of the local community?

## 2. Materials and Methods

This research utilized data from the 2023 Korea Community Health Survey (KCHS), conducted by the Korean government. Using data from the 2019 KCHS, Chang [15] analyzed differences in SRH and physical activity according to educational levels, emphasizing the importance of school physical education. Expanding the focus from school physical education to lifelong physical education, this study explores the conditions necessary for its establishment. Specifically, it examines physical activity and SRH by considering satisfaction with the physical environment of the community, a key factor influencing lifelong physical education.

### 2.1. Participants

The 2023 KCHS used in this study was conducted in collaboration with the Korea Disease Control and Prevention Agency, local governments, health centers, and designated universities, with the aim of producing regional health statistics, standardizing survey indicators, and establishing a standardized survey system [17]. The KCHS, conducted annually since 2008, recently released raw data for the 2023 survey.

The target population included adults aged 19 and older based on the Korean resident registration system. The first stage of sample selection was performed using probability proportional to size systematic sampling, considering the number of households by housing type for each sample point. The second stage involved systematic sampling based on the number of households at sample points. Overall, 231,752 surveys were conducted based on the selected sample, with the characteristics of the study subjects detailed in Table 1.

### 2.2. Items and Measurements

The survey was conducted from 16 May to 31 July 2023, using computer-assisted personal interviewing, with trained interviewers visiting selected households and conducting one-on-one interviews. To verify the data and ensure quality control, 13% of the completed surveys were resampled for telephone verification. Discrepancies, if any occurred, were rectified, and the results were reported to the Korea Disease Control and Prevention Agency.

The data used in this study comprised household and individual surveys, including 145 questions. The household survey covered five areas, including household type and income. The individual survey addressed 16 areas, including drinking, smoking, physical activity, SRH, and the social physical environment. The physical environment of local communities was considered part of the social physical environment, along with social networks and social activities.

Since the raw data from the KCHS did not include private identifiers, such as home address, telephone number, or social security number, ethical approval was not required. According to Article 2, Paragraph 2 of the Enforcement Rule of the Bioethics and Safety Act of South Korea, the KCHS is not considered to be human subjects research and is, therefore, exempt from Institutional Review Board review.

The physical environment was assessed based on public satisfaction with safety, the natural and living environments, public transportation, and medical services. The study variables included subjective health status and physical activity, with subvariables for physical activity encompassing the duration of vigorous and moderate physical activity, walking, and flexibility exercises. The study variables are explained in Appendix A.

### 2.3. Data Processing

The collected data were transmitted in real time to a central server by investigators using tablet PCs, with local officials monitoring the progress. The finalized statistics are published annually on the Korea Community Health Survey website (https://chs.kdca.go.kr/ (accessed on 4 April 2024)).

**Figure 1:**
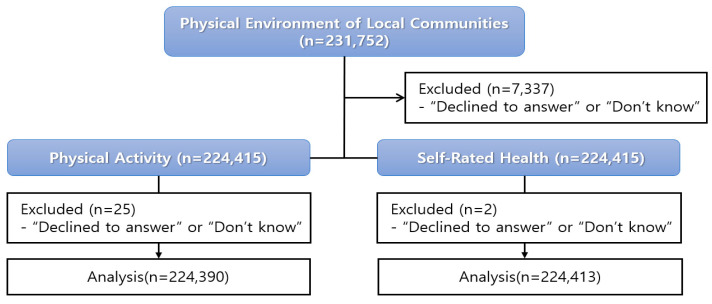
Flow diagram.

To explore the differences in physical activity and SRH according to satisfaction with the physical environment of local communities, this study used the statistical program SPSS Windows Version 18.0. Multivariate analysis of variance (MANOVA) and univariate analysis of variance (ANOVA) were performed to calculate the means and standard deviations. A MANOVA was conducted to examine the main effects and interaction effects of satisfaction with the physical environment of local communities on the four physical activity factors. Additionally, an ANOVA was performed to assess the main effects of satisfaction with the physical environment and the interaction effects between factors on SRH. The data processing flow diagram is shown in Figure 1.

First, 7337 cases were excluded from the survey on satisfaction with the physical environment of local communities where respondents answered “refuse to respond” or “don’t know”. To explore differences in physical activity based on satisfaction with the physical environment of local communities, 25 cases where respondents answered “refuse to respond” or “don’t know” were excluded. Additionally, two cases were excluded from the data collected to explore differences in self-rated health based on satisfaction with the physical environment of local communities. Statistical significance levels were set at *p* < 0.05.

## 3. Results

### 3.1. Main Effects of Physical Activity According to Satisfaction with the Physical Environment of Local Communities

To verify the main and interaction effects of satisfaction with factors related to the physical environment of local communities on physical activity factors, such as the number of days of vigorous physical activity, moderate physical activity, walking, and flexibility exercises, MANOVA was conducted.

The results showed significant differences in the number of days of moderate physical activity and walking depending on satisfaction with safety levels; vigorous physical activity and walking depending on satisfaction with the natural environment; moderate physical activity, walking, and flexibility exercises depending on satisfaction with medical services; and all physical activity factors depending on satisfaction with the living environment and public transportation (*p* < 0.05).

Regarding the interaction effects between factors, significant differences were observed in the number of days of vigorous physical activity, moderate physical activity, and flexibility exercises depending on satisfaction with both safety levels and natural environment; flexibility exercises depending on satisfaction with both safety levels and public transportation; moderate physical activity and flexibility exercises depending on satisfaction with both safety levels and medical services; vigorous physical activity, moderate physical activity, and flexibility exercises depending on satisfaction with both the natural living environment; moderate physical activity depending on satisfaction with both the natural environment and medical services; and walking depending on satisfaction with both the living environment and public transportation (*p* < 0.05).

Additionally, significant differences were found in the number of days of moderate physical activity depending on satisfaction with both the natural and living environment, medical services, and public transportation; flexibility exercises depending on satisfaction with both the natural environment and public transportation and medical services and public transportation; and vigorous physical activity depending on satisfaction with safety levels, the natural and living environment, and medical services. Furthermore, significant differences were also found in the number of days of walking depending on satisfaction with the natural and living environment, public transportation, and medical services (*p* < 0.05) (See Table 2).

### 3.2. Interaction Effects of Physical Activity According to Satisfaction with the Physical Environment of Local Communities

Bonferroni’s multiple comparison test was conducted for variables where the main effect of satisfaction with the physical environment of local communities on physical activity was found to be significant. The results showed that the number of days of vigorous physical activity was higher in groups dissatisfied with the natural and living environment and public transportation, compared to those who were satisfied. The number of days of moderate physical activity was higher in groups satisfied with safety levels compared to those who were dissatisfied. However, for living environment, public transportation, and medical services, the dissatisfied group had higher activity levels than the satisfied group. The number of days of walking was higher in groups satisfied with public transportation and medical services, compared to those who were dissatisfied. Meanwhile, for safety levels and natural and living environment, the dissatisfied group reported walking for a higher number of days than the satisfied group. Lastly, the number of days of flexibility exercises was higher in groups satisfied with public transportation and medical services, but in the living environment, the dissatisfied group showed higher activity levels than the satisfied group (see Table 3).

### 3.3. Main Effects of Self-Rated Health Based on Satisfaction with the Physical Environment of Local Communities

An ANOVA was conducted to examine the main effects of satisfaction with the physical environment of local communities and the interaction effects between factors on SRH.

The results showed that the main effects on SRH were significant for safety levels (F = 37.167, *p* < 0.05), living environment (F = 11.813, *p* < 0.05), and medical services (F = 31.783, *p* < 0.05). However, the main effects of the natural environment and public transportation were not significant. Regarding the interaction effects between factors, significant interactions were found between safety levels and the natural environment (F = 11.125, *p* < 0.05); the natural and living environment (F = 10.31, *p* < 0.05); and the natural environment, public transportation, and medical services (F = 5.934, *p* < 0.05) at the 5% significance level. Additionally, the interaction between safety levels, the natural environment, and medical services (F = 3.020, *p* < 0.1) was significant at the 10% significance level (see Table 4).

### 3.4. Interaction Effects of Self-Rated Health Based on Satisfaction with the Physical Environment of Local Communities

The results of ANOVA showed that the main effects of satisfaction with the physical environment of local communities on SRH were significant for safety levels, living environment, and medical services. According to Bonferroni’s multiple comparison test, the safety level satisfaction group (M = 3.223) had higher SRH than the dissatisfaction group (M = 3.174). Similarly, individuals satisfied with medical services (M = 3.222) had higher SRH than those who were dissatisfied (M = 3.175). Meanwhile, for the living environment, the group that was dissatisfied (M = 3.213) had higher SRH than the satisfied group (M = 3.185) (see Table 5).

## 4. Discussion

This study utilized data from the 2023 KCHS to explore differences in physical activity and SRH according to satisfaction with the physical environment of local communities. The study provides foundational data to understand the impact of social and physical environments on health and physical activity, extending beyond school physical education within public education. Additionally, the study sought to identify ways in which lifelong physical education can contribute to improving quality of life. 

### 4.1. Physical Activity According to Satisfaction with the Physical Environment of Local Communities

The findings are significant in that they provide a multifaceted analysis of the impact of satisfaction with the physical environment of local communities on physical activity levels. The finding that there are significant differences in various physical activity factors depending on environmental satisfaction is particularly important. The analysis of the impact of physical environmental factors, such as safety levels, natural and living environment, public transportation, and medical services on physical activity offers important implications. These findings suggest the need to improve environmental factors for promoting physical activity; although, environmental satisfaction alone does not predict activity levels.

Related studies have also explored the impact of community environment on physical activity in various ways. Cerin et al. [18] emphasized that various environmental factors globally influence physical activity and reported that the physical environment in urban areas can positively influence physical activity. Similarly, Sallis et al. [19], in a study of 20 cities worldwide, argued that a safe and well-designed urban environment plays a crucial role in promoting physical activity. However, in the results of this study, some environmental factors showed that the group that was dissatisfied had higher levels of physical activity than the group that was satisfied. This suggests that dissatisfaction with the environment could actually serve as a factor to promote physical activity. For example, a study by Sugiyama et al. [20] demonstrated that a walkable environment does not necessarily lead to higher physical activity, and that individual social and psychological factors could also play a crucial role.

The finding that the number of days of vigorous physical activity was higher in groups dissatisfied with the natural and living environment and public transportation suggests that dissatisfaction with specific environments could actually promote active participation in physical activity. This may imply that physical activity is used as an alternative activity to relieve stress caused by dissatisfaction or to overcome daily inconveniences [21]. Meanwhile, for moderate physical activity, the group satisfied with safety levels recorded more days of activity, indicating that a safe environment could be a major factor in promoting participation in moderate physical activity [22]. These results suggest that a safe environment provides a psychological safety net for physical activity, which in turn increases participation [23].

Additionally, the study presents paradoxical results, suggesting that environmental dissatisfaction could have a positive impact on physical activity levels, offering a new perspective on policy approaches for promoting physical activity. For instance, it highlights the need to develop various alternative programs that could encourage physical activity even in environments with dissatisfaction factors. Such programs may include challenging activities to overcome dissatisfaction or strategies that motivate community members to overcome environmental constraints themselves [24].

The implication is that not only is there a need for policy interventions that can encourage physical activity in environments with dissatisfaction factors, but there is also a need to promote physical activity by improving the physical environment of communities. This suggests that increasing environmental satisfaction is not the only way to enhance physical activity, and that strategies for promoting physical activity in various environmental contexts are required [25]. For example, in cases where dissatisfaction with elements such as the living environment or public transportation has a positive impact on physical activity, it may be important to develop alternative physical activity programs that help overcome these environmental constraints [26]. In this context, further research is needed to clarify the causes of how environmental dissatisfaction promotes physical activity. Understanding the social and psychological mechanisms by which dissatisfaction acts as a motivator will be a key task for future research [27].

These findings emphasize that when formulating public policies aimed at promoting physical activity, a multifaceted approach that considers dissatisfaction factors is necessary, rather than focusing solely on environmental improvements. In addition to improving the quality of the physical environment within communities, there is a need for strategic interventions that could turn perceived environmental dissatisfaction into opportunities to promote physical activity [28]. A comprehensive approach that takes into account the impact of social inequality and economic factors on physical activity and health is required in the policy-making process [29]. Additionally, tailored policies that support individuals from diverse social backgrounds to promote physical activity in different environments are needed.

### 4.2. Self-Rated Health According to Satisfaction with the Physical Environment of Local Communities

This study provides a multifaceted analysis of how satisfaction with the physical environment of local communities influences SRH, highlighting the significant impact of specific environmental factors on subjective health. In particular, the analysis of the effects of physical environmental factors, such as safety levels, living environment, and medical services, on subjective health offers important implications when compared to previous studies. These findings suggest that improving SRH requires not only the enhancement of the physical environment, but also the consideration of psychological factors related to individuals’ satisfaction with their environment.

Previous studies have also explored the impact of the physical environment in communities on subjective health in various ways. Regarding safety levels, some studies have reported that the safer a community is perceived to be, the more positively residents evaluate their own health [30]. Additionally, research consistently shows that higher safety levels reduce psychological stress, thereby improving SRH [31]. Other studies have shown that a well-established social safety net within a community could positively influence individual health perceptions [32]. Conversely, low safety levels have been argued to negatively impact both physical and mental health [33]. Particularly among the elderly, low safety levels have been found to negatively affect health [34]. These studies are consistent with the findings of this research, which show that satisfaction with safety levels influences SRH.

The finding that the natural environment does not significantly impact SRH contrasts with previous research. Earlier studies have reported that the natural environment positively influences health perceptions [11,12,35,36,37]. This discrepancy may be attributed to the unique regional characteristics of Korea, where many people live in densely populated, urban areas driven by urbanization. Therefore, the impact of the natural environment on health perceptions in daily life may be limited. Kim et al. [38] argued that, in Korea, social capital and economic stability have a greater influence on SRH than the natural environment. Similarly, a study by Kim and Cho [39] found that the living environment and social support networks had a greater impact on health perceptions than the natural environment. Research has also shown that factors such as access to public transportation, convenience facilities, residential economic stability, and medical services have a more significant impact on SRH than the natural environment [40,41]. This suggests that economic and social determinants of health play a stronger role than the natural environment in Korea.

The finding that satisfaction with the living environment positively influences SRH is consistent with previous research. Studies have shown that the higher the quality of the living environment, the more positively residents evaluate their health [42]. Other research has argued that a clean and well-maintained environment is associated with higher SRH [43]. Additionally, the quality of the living environment, particularly access to nearby facilities and services, and the quality of local shops, has been reported to positively impact SRH [44]. Middle-aged and elderly individuals living in areas with good physical environments are also more likely to report higher SRH [45]. The positive impact of green spaces on SRH can also be understood within this context [46].

However, it is worth noting that, in this study, the group dissatisfied with their living environment actually reported higher SRH. This suggests that dissatisfaction with the living environment may motivate individuals to improve their health perceptions. The Poor People’s Campaign [47] pointed out that a dissatisfactory living environment could actually promote individual efforts and determination to improve health. This reflects a situation where dissatisfaction with the living environment leads to more proactive self-management or behavior changes aimed at maintaining and improving health.

The finding that satisfaction with public transportation does not significantly impact SRH contrasts with previous research. Earlier studies have reported that access to public transportation significantly influences physical activity and subjective health [48,49,50,51,52]. However, it can be interpreted that, in Korea, where the public transportation system is already well-developed, the impact of public transportation satisfaction on health may not be significant [13,14]. Additionally, studies suggesting that social relationships and workplace environments have a greater impact on self-rated health than public transportation can help explain these results [53].

The finding that satisfaction with medical services positively influences SRH is consistent with previous research. A representative study showed that improved access to medical services leads to individuals evaluating their health more positively [54]. Other research has also found that individuals residing in areas with good medical services tend to evaluate their health more positively [55]. Additionally, studies have consistently shown that higher access to medical services encourages residents to evaluate their health more positively [56]. The tendency for individuals to evaluate their health more positively when they have a trusting relationship with their primary care physician can also be understood in this context [57].

In conclusion, this study provides a comprehensive analysis of how the physical environment of local communities influences SRH, revealing that high environmental satisfaction does not always correlate with higher SRH. This emphasizes the need to not only improve the physical environment but also provide psychological and social support that could transform environmental dissatisfaction into a positive motivator for enhancing subjective health. An alternative approach to improving health in environments with dissatisfaction factors is required. This suggests the necessity of a personalized approach that considers environmental satisfaction when formulating policies aimed at promoting health. Furthermore, in Korea, where rapid urbanization has driven regional imbalances, and economic and social factors are perceived to have a greater impact on health [38], it is advisable to analyze the sociocultural factors influencing health in each region and identify and improve the physical environments that require priority attention. In this context, future research should comprehensively explore the various environmental and psychological factors that influence SRH. This will contribute to more effectively establishing community health promotion strategies.

### 4.3. Limitations and Scope for Further Research

Several limitations of this study should be acknowledged, and suggestions for future research addressing these limitations are proposed. First, this study was a large-scale project targeting a broad range of participants. Future studies should stratify and compare data by sex, age, and geographic region. Second, this study utilized large-scale national public data, which typically include numerous variables. Future research should compare variables across groups based on the physical environment or incorporate additional factors beyond the physical environment. Third, the physical activity variables in this study included work-related activity, which differs from the concept of leisure physical activity. Future studies should explore work-related activity and leisure physical activity separately. Fourth, this study examined differences in physical activity and SRH based on satisfaction with the local community’s physical environment without considering other variables. Future research should investigate differences in variables based on participants’ health conditions, such as hypertension and diabetes. Lastly, this study employed MANOVA and ANOVA to analyze differences. Future research should consider using multivariate regression analysis or other methods to achieve more precise results and explore the relationships between variables through qualitative approaches.

## 5. Conclusions

This study utilized data from the 2023 KCHS to explore differences in physical activity and SRH according to satisfaction with the physical environment of local communities, targeting 231,752 adults aged 19 and older. The aim was to provide foundational data for policy efforts to identify and address the causes of increasingly deepening regional health disparities. The results are as follows:

First, regarding physical activity according to satisfaction with the physical environment of local communities, significant differences were observed based on satisfaction with safety levels in the number of days of moderate physical activity and walking; vigorous physical activity and walking based on satisfaction with the natural environment; moderate physical activity, walking, and flexibility exercises based on satisfaction with medical services; and all physical activity factors based on satisfaction with the living environment and public transportation.

The interaction effect between factors revealed significant differences in various physical activities when certain factors were aligned. In multiple comparisons, the results consistently demonstrated that more physical activity was performed when participants were satisfied with the local community’s physical environment and when they were dissatisfied with the physical environment.

Second, concerning SRH according to satisfaction with the physical environment of local communities, significant differences were found in safety levels, living environment, and medical services. The groups satisfied with safety levels and medical services reported higher SRH than the dissatisfied groups; whereas, for the living environment, the dissatisfied group reported higher SRH than the satisfied group.

In the multiple comparisons, the group satisfied with the safety and medical services reported higher SRH than the dissatisfied group; however, the opposite result was observed for the living environment.

In summary, satisfaction with the physical environment of local communities was found to have different impacts on physical activity and SRH depending on the factors involved. Satisfaction with certain aspects of the physical environment positively impacted both physical activity and SRH. This suggests the need for practical efforts to reduce disparities in the physical environment between regions and to increase residents’ satisfaction. Tailored strategies for each region’s specific factors are necessary to create a physical environment that bridges regional health disparities. Furthermore, there is a need to reflect on how national-level health policies have relied on school physical education and have not devoted sufficient policy efforts in addressing the health issues of adults beyond their student years. The significant differences in satisfaction ratios among the factors related to the physical environment of local communities indicate that social infrastructure has not been adequately established. It is imperative to focus on the goal of lifelong physical education across all age groups and to work toward creating the necessary physical environments in local communities.

## Figures and Tables

**Table 1:** General characteristics of the participants.

Variables	Categories																												
	N (%)																												
Gender	Male															Female													
	105,754 (45.6)															125,998 (54.4)													
Age (years)	19~29					30~39					40~49					50~59				60~69					>70				
	21,540 (9.3)					24,339 (10.5)					33,816 (14.6)					42,753 (18.4)				51,853 (22.4)					57,451 (24.8)				
Satisfaction with the physical environment of local communities	Safety Levels						Natural Environment							Living Environment				Public Transportation						Medical Services					
	Yes	No	Refuse to respond		Don’t know		Yes		No	Refuse to respond		Don’t know		Yes	No	Refuse to respond	Don’t know	Yes		No	Refuse to respond		Don’t know	Yes		No		Refuse to respond	Don’t know
	198,811 (85.8)	30,124 (13.0)	34 (0.0)		2783 (1.2)		192,404 (83.0)		38,277 (16.5)	27 (0.0)		1044 (0.5)		197,975 (85.4)	32,943 (14.2)	26 (0.0)	808 (0.3)	160,905 (69.4)		66,952 (28.9)	29 (0.0)		3866 (1.7)	169,532 (73.2)		60,417 (26.1)		30 (0.0)	1773 (0.8)
Self-rated health	Very good				Good					Average					Poor		Very poor				Refuse to respond					Don’t know			
	12,825 (5.5)				75,341 (32.5)					98,935 (42.7)					36,125 (15.6)		8524 (3.7)				0 (0.0)					2 (0.0)			
Number of days of vigorous physical activity (days/week)	None		1				2			3				4		5		6			7			Refuse to respond				Don’t know	
	174,829 (75.4)		12,533 (5.4)				11,877 (5.1)			11,962 (5.2)				4855 (2.1)		8115 (3.5)		2249 (1.0)			5321 (2.3)			2 (0.0)				9 (0.0)	
Number of days of moderate intensity physical activity (days/week)	None		1				2			3				4		5		6			7			Refuse to respond				Don’t know	
	146,678 (63.3)		11,624 (5.0)				16,034 (6.9)			18,347 (7.9)				6705 (2.9)		14,808 (6.4)		4095 (1.8)			13,448 (5.8)			1 (0.0)				12 (0.0)	
Number of days of walking (days/week)	None		1				2			3				4		5		6			7			Refuse to respond				Don’t know	
	40,183 (17.3)		8743 (3.8)				16,794 (7.2)			26,203 (11.3)				13,261 (5.7)		35,546 (15.3)		11,126 (4.8)			79,888 (34.5)			1 (0.0)				7 (0.0)	
Number of days of flexibility exercise (days/week)	None			1				2					3			4			>5			Refuse to respond					Don’t know		
	103,048 (44.5)			10,610 (4.6)				18,455 (8.0)					25,355 (10.9)			9455 (4.1)			64,817 (28.0)			0 (0.0)					12 (0.0)		
Hypertension diagnosis experience	Yes							No								Refuse to respond						Don’t know							
	72,594 (31.3)							159,144 (68.7)								2 (0.0)						12 (0.0)							
Diabetes diagnosis experience	Yes							No								Refuse to respond						Don’t know							
	31,725 (13.7)							200,011 (86.3)								2 (0.0)						14 (0.0)							

Tested using frequency analysis.

**Table 2:** Physical activity according to satisfaction with the physical environment of local communities (multivariate analysis of variance).

Factor	Dependent Variable	Sum of Squares	DF	Mean Square	F	Significance Probability
Safety Levels	Number of days of vigorous physical activity	0.904	1	0.904	1.480	0.224
	Number of days of moderate intensity physical activity	35.906	1	35.906	39.609 ***	0.000
	Number of days of walking	6.604	1	6.604	7.468 **	0.006
	Number of days of flexibility exercise	2.846	1	2.846	2.906	0.088
Natural Environment	Number of days of vigorous physical activity	5.445	1	5.445	8.910 **	0.003
	Number of days of moderate intensity physical activity	0.003	1	0.003	0.003	0.957
	Number of days of walking	22.964	1	22.964	25.967 ***	0.000
	Number of days of flexibility exercise	3.521	1	3.521	3.595	0.058
Living Environment	Number of days of vigorous physical activity	31.967	1	31.967	52.312 ***	0.000
	Number of days of moderate intensity physical activity	7.561	1	7.561	8.341 **	0.004
	Number of days of walking	25.381	1	25.381	28.700 ***	0.000
	Number of days of flexibility exercise	6.871	1	6.871	7.016 **	0.008
Public Transportation	Number of days of vigorous physical activity	3.271	1	3.271	5.352 *	0.021
	Number of days of moderate intensity physical activity	4.108	1	4.108	4.531 *	0.033
	Number of days of walking	98.143	1	98.143	110.981 ***	0.000
	Number of days of flexibility exercise	21.064	1	21.064	21.509 ***	0.000
Medical Services	Number of days of vigorous physical activity	0.295	1	0.295	0.483	0.487
	Number of days of moderate intensity physical activity	9.530	1	9.530	10.513 *	0.001
	Number of days of walking	135.635	1	135.635	153.376 ***	0.000
	Number of days of flexibility exercise	24.499	1	24.499	25.016 ***	0.000
Safety Levels * Natural Environment	Number of days of vigorous physical activity	5.785	1	5.785	9.466 **	0.002
	Number of days of moderate intensity physical activity	9.336	1	9.336	10.298 **	0.001
	Number of days of walking	1.726	1	1.726	1.952	0.162
	Number of days of flexibility exercise	10.153	1	10.153	10.367 **	0.001
Safety Levels * Living Environment	Number of days of vigorous physical activity	0.937	1	0.937	1.534	0.216
	Number of days of moderate intensity physical activity	1.833	1	1.833	2.022	0.155
	Number of days of walking	2.456	1	2.456	2.777	0.096
	Number of days of flexibility exercise	3.470	1	3.470	3.543	0.060
Safety Levels * Public Transportation	Number of days of vigorous physical activity	0.195	1	0.195	0.320	0.572
	Number of days of moderate intensity physical activity	1.785	1	1.785	1.969	0.161
	Number of days of walking	0.142	1	0.142	0.161	0.688
	Number of days of flexibility exercise	3.818	1	3.818	3.898 *	0.048
Safety Levels * Medical Services	Number of days of vigorous physical activity	0.941	1	0.941	1.540	0.215
	Number of days of moderate intensity physical activity	3.631	1	3.631	4.006 *	0.045
	Number of days of walking	0.823	1	0.823	0.930	0.335
	Number of days of flexibility exercise	8.442	1	8.442	8.620 **	0.003
Natural Environment * Living Environment	Number of days of vigorous physical activity	4.945	1	4.945	8.092 **	0.004
	Number of days of moderate intensity physical activity	6.451	1	6.451	7.116 **	0.008
	Number of days of walking	0.984	1	0.984	1.113	0.291
	Number of days of flexibility exercise	7.050	1	7.050	7.199 **	0.007
Natural Environment * Public Transportation	Number of days of vigorous physical activity	1.043	1	1.043	1.708	0.191
	Number of days of moderate intensity physical activity	2.253	1	2.253	2.485	0.115
	Number of days of walking	2.107	1	2.107	2.382	0.123
	Number of days of flexibility exercise	1.129	1	1.129	1.153	0.283
Natural Environment * Medical Services	Number of days of vigorous physical activity	1.852	1	1.852	3.031	0.082
	Number of days of moderate intensity physical activity	4.501	1	4.501	4.965 *	0.026
	Number of days of walking	1.179	1	1.179	1.333	0.248
	Number of days of flexibility exercise	0.142	1	0.142	0.145	0.704
Living Environment * Public Transportation	Number of days of vigorous physical activity	0.177	1	0.177	0.289	0.591
	Number of days of moderate intensity physical activity	1.378	1	1.378	1.520	0.218
	Number of days of walking	4.111	1	4.111	4.649 *	0.031
	Number of days of flexibility exercise	3.034	1	3.034	3.098	0.078
Living Environment * Medical Services	Number of days of vigorous physical activity	0.305	1	0.305	0.498	0.480
	Number of days of moderate intensity physical activity	0.259	1	0.259	0.286	0.593
	Number of days of walking	0.378	1	0.378	0.427	0.514
	Number of days of flexibility exercise	0.761	1	0.761	0.777	0.378
Public Transportation * Medical Services	Number of days of vigorous physical activity	0.876	1	0.876	1.434	0.231
	Number of days of moderate intensity physical activity	0.070	1	0.070	0.077	0.782
	Number of days of walking	0.319	1	0.319	0.361	0.548
	Number of days of flexibility exercise	0.018	1	0.018	0.019	0.891
Safety Levels * Natural Environment * Living Environment	Number of days of vigorous physical activity	0.184	1	0.184	0.302	0.583
	Number of days of moderate intensity physical activity	2.326	1	2.326	2.566	0.109
	Number of days of walking	0.091	1	0.091	0.102	0.749
	Number of days of flexibility exercise	0.603	1	0.603	0.616	0.432
Safety Levels * Natural Environment * Public Transportation	Number of days of vigorous physical activity	0.130	1	0.130	0.213	0.644
	Number of days of moderate intensity physical activity	0.062	1	0.062	0.069	0.793
	Number of days of walking	0.002	1	0.002	0.002	0.966
	Number of days of flexibility exercise	0.001	1	0.001	0.001	0.982
Safety Levels * Natural Environment * Medical Services	Number of days of vigorous physical activity	1.184	1	1.184	1.937	0.164
	Number of days of moderate intensity physical activity	0.173	1	0.173	0.191	0.662
	Number of days of walking	1.651	1	1.651	1.867	0.172
	Number of days of flexibility exercise	1.569	1	1.569	1.602	0.206
Safety Levels * Living Environment * Public Transportation	Number of days of vigorous physical activity	0.006	1	0.006	0.010	0.919
	Number of days of moderate intensity physical activity	0.092	1	0.092	0.102	0.750
	Number of days of walking	0.826	1	0.826	0.935	0.334
	Number of days of flexibility exercise	0.920	1	0.920	0.940	0.332
Safety Levels * Living Environment * Medical Services	Number of days of vigorous physical activity	0.147	1	0.147	0.241	0.624
	Number of days of moderate intensity physical activity	0.011	1	0.011	0.012	0.912
	Number of days of walking	0.217	1	0.217	0.245	0.620
	Number of days of flexibility exercise	0.760	1	0.760	0.776	0.378
Safety Levels * Public Transportation * Medical Services	Number of days of vigorous physical activity	0.213	1	0.213	0.348	0.555
	Number of days of moderate intensity physical activity	0.009	1	0.009	0.010	0.919
	Number of days of walking	0.140	1	0.140	0.158	0.691
	Number of days of flexibility exercise	0.092	1	0.092	0.094	0.759
Natural Environment * Living Environment * Public Transportation	Number of days of vigorous physical activity	1.865	1	1.865	3.051	0.081
	Number of days of moderate intensity physical activity	3.119	1	3.119	3.440	0.064
	Number of days of walking	0.202	1	0.202	0.229	0.632
	Number of days of flexibility exercise	0.675	1	0.675	0.689	0.407
Natural Environment * Living Environment * Medical Services	Number of days of vigorous physical activity	1.902	1	1.902	3.113	0.078
	Number of days of moderate intensity physical activity	4.827	1	4.827	5.324 *	0.021
	Number of days of walking	0.160	1	0.160	0.181	0.671
	Number of days of flexibility exercise	0.384	1	0.384	0.392	0.531
Natural Environment * Public Transportation * Medical Services	Number of days of vigorous physical activity	1.135	1	1.135	1.857	0.173
	Number of days of moderate intensity physical activity	1.226	1	1.226	1.352	0.245
	Number of days of walking	0.869	1	0.869	0.983	0.321
	Number of days of flexibility exercise	6.551	1	6.551	6.689 *	0.010
Living Environment * Public Transportation * Medical Services	Number of days of vigorous physical activity	0.025	1	0.025	0.041	0.840
	Number of days of moderate intensity physical activity	2.311	1	2.311	2.550	0.110
	Number of days of walking	8.144	1	8.144	9.210 **	0.002
	Number of days of flexibility exercise	1.169	1	1.169	1.194	0.275
Safety Levels * Natural Environment * Living Environment * Public Transportation	Number of days of vigorous physical activity	0.179	1	0.179	0.293	0.588
	Number of days of moderate intensity physical activity	0.026	1	0.026	0.029	0.865
	Number of days of walking	2.427	1	2.427	2.744	0.098
	Number of days of flexibility exercise	0.247	1	0.247	0.252	0.616
Safety Levels * Natural Environment * Living Environment * Medical Services	Number of days of vigorous physical activity	2.549	1	2.549	4.171 *	0.041
	Number of days of moderate intensity physical activity	1.441	1	1.441	1.590	0.207
	Number of days of walking	1.489	1	1.489	1.684	0.194
	Number of days of flexibility exercise	0.549	1	0.549	0.561	0.454
Safety Levels * Natural Environment * Public Transportation * Medical Services	Number of days of vigorous physical activity	1.323	1	1.323	2.165	0.141
	Number of days of moderate intensity physical activity	0.218	1	0.218	0.241	0.624
	Number of days of walking	0.646	1	0.646	0.730	0.393
	Number of days of flexibility exercise	0.226	1	0.226	0.231	0.631
Safety Levels * Living Environment * Public Transportation * Medical Services	Number of days of vigorous physical activity	0.361	1	0.361	0.591	0.442
	Number of days of moderate intensity physical activity	0.392	1	0.392	0.432	0.511
	Number of days of walking	2.302	1	2.302	2.603	0.107
	Number of days of flexibility exercise	0.894	1	0.894	0.913	0.339
Natural Environment * Living Environment * Public Transportation * Medical Services	Number of days of vigorous physical activity	0.024	1	0.024	0.040	0.842
	Number of days of moderate intensity physical activity	0.235	1	0.235	0.260	0.610
	Number of days of walking	3.868	1	3.868	4.374 *	0.036
	Number of days of flexibility exercise	2.343	1	2.343	2.393	0.122
Safety Levels * Natural Environment * Living Environment * Public Transportation * Medical Services	Number of days of vigorous physical activity	0.633	1	0.633	1.036	0.309
	Number of days of moderate intensity physical activity	0.052	1	0.052	0.058	0.810
	Number of days of walking	0.474	1	0.474	0.536	0.464
	Number of days of flexibility exercise	0.034	1	0.034	0.035	0.853
Error Term	Number of days of vigorous physical activity	137,099.018	224,358	0.611		
	Number of days of moderate intensity physical activity	203,385.371	224,358	0.907		
	Number of days of walking	198,406.535	224,358	0.884		
	Number of days of flexibility exercise	219,724.016	224,358	0.979		

* *p* < 0.05, ** *p* < 0.01, *** *p* < 0.001, tested using MANOVA.

**Table 3:** Comparison of estimated means of self-rated health according to satisfaction with the physical environment of local communities.

Dependent Variable	Physical Environment of Local Communities		Mean	Standard Error
Number of days of vigorous physical activity	Natural Environment	Satisfied	0.463 ^a^	0.005
		Dissatisfied	0.484 ^b^	0.005
	Living Environment	Satisfied	0.448 ^a^	0.004
		Dissatisfied	0.499 ^b^	0.006
	Public Transportation	Satisfied	0.466 ^a^	0.005
		Dissatisfied	0.482 ^b^	0.005
Number of days of moderate intensity physical activity	Safety Levels	Satisfied	0.731 ^b^	0.005
		Dissatisfied	0.677 ^a^	0.007
	Living Environment	Satisfied	0.691 ^a^	0.005
		Dissatisfied	0.716 ^b^	0.007
	Public Transportation	Satisfied	0.695 ^a^	0.006
		Dissatisfied	0.713 ^b^	0.006
	Medical Services	Satisfied	0.690 ^a^	0.006
		Dissatisfied	0.718 ^b^	0.006
Number of days of walking	Safety Levels	Satisfied	1.822 ^a^	0.005
		Dissatisfied	1.845 ^b^	0.007
	Natural Environment	Satisfied	1.812 ^a^	0.006
		Dissatisfied	1.855 ^b^	0.006
	Living Environment	Satisfied	1.811 ^a^	0.005
		Dissatisfied	1.856 ^b^	0.007
	Public Transportation	Satisfied	1.878 ^b^	0.006
		Dissatisfied	1.789 ^a^	0.006
	Medical Services	Satisfied	1.886 ^b^	0.006
		Dissatisfied	1.781 ^a^	0.006
Number of days of flexibility exercise	Living Environment	Satisfied	1.075 ^a^	0.006
		Dissatisfied	1.098 ^b^	0.007
	Public Transportation	Satisfied	1.107 ^b^	0.006
		Dissatisfied	1.066 ^a^	0.006
	Medical Services	Satisfied	1.109 ^b^	0.006
		Dissatisfied	1.064 ^a^	0.007

Bonferroni: a < b, tested using MANOVA.

**Table 4:** Self-rated health according to satisfaction with the physical environment of local communities.

Variable	Sum of Squares	DF	Mean Square	F	Significance Probability
Safety Levels	29.977	1	29.977	37.167 ***	0.000
Natural Environment	0.894	1	0.894	1.109	0.292
Living Environment	9.527	1	9.527	11.813 **	0.001
Public Transportation	0.045	1	0.045	0.055	0.814
Medical Services	25.635	1	25.635	31.783 ***	0.000
Safety Levels * Natural Environment	8.973	1	8.973	11.125 **	0.001
Safety Levels * Living Environment	0.067	1	0.067	0.083	0.773
Safety Levels * Public Transportation	0.343	1	0.343	0.425	0.515
Safety Levels * Medical Services	0.038	1	0.038	0.048	0.827
Natural Environment * Living Environment	8.318	1	8.318	10.312 **	0.001
Natural Environment * Public Transportation	0.165	1	0.165	0.205	0.651
Natural Environment * Medical Services	0.198	1	0.198	0.245	0.621
Living Environment * Public Transportation	0.541	1	0.541	0.671	0.413
Living Environment * Medical Services	0.005	1	0.005	0.006	0.936
Public Transportation * Medical Services	0.001	1	0.001	0.001	0.976
Safety Levels * Natural Environment * Living Environment	0.654	1	0.654	0.811	0.368
Safety Levels * Natural Environment * Public Transportation	0.579	1	0.579	0.718	0.397
Safety Levels * Natural Environment * Medical Services	2.436	1	2.436	3.020	0.082
Safety Levels * Living Environment * Public Transportation	0.515	1	0.515	0.638	0.424
Safety Levels * Living Environment * Medical Services	1.051	1	1.051	1.303	0.254
Safety Levels * Public Transportation * Medical Services	0.247	1	0.247	0.306	0.580
Natural Environment * Living Environment * Public Transportation	0.026	1	0.026	0.033	0.857
Natural Environment * Living Environment * Medical Services	1.430	1	1.430	1.773	0.183
Natural Environment * Public Transportation * Medical Services	4.786	1	4.786	5.934 *	0.015
Living Environment * Public Transportation * Medical Services	0.247	1	0.247	0.306	0.580
Safety Levels * Natural Environment * Living Environment * Public Transportation	0.083	1	0.083	0.103	0.748
Safety Levels * Natural Environment * Living Environment * Medical Services	0.292	1	0.292	0.362	0.548
Safety Levels * Natural Environment * Public Transportation * Medical Services	0.049	1	0.049	0.061	0.804
Safety Levels * Living Environment * Public Transportation * Medical Services	1.230	1	1.230	1.525	0.217
Natural Environment * Living Environment * Public Transportation * Medical Services	0.028	1	0.028	0.035	0.852
Safety Levels * Natural Environment * Living Environment * Public Transportation * Medical Services	0.055	1	0.055	0.068	0.794
Error	180,974.268	224,381	0.807		

* *p* < 0.05, ** *p* < 0.01, *** *p* < 0.001, tested using ANOVA.

**Table 5:** Comparison of estimated means of self-rated health according to satisfaction with the physical environment of local communities.

Dependent Variable	Physical Environment of Local Communities		Mean	Standard Error
Self-rated health	Safety Levels	Satisfied	3.223 ^b^	0.005
		Dissatisfied	3.174 ^a^	0.007
	Living Environment	Satisfied	3.185 ^a^	0.005
		Dissatisfied	3.213 ^b^	0.006
	Medical Services	Satisfied	3.222 ^b^	0.005
		Dissatisfied	3.175 ^a^	0.006

Bonferroni: a < b, tested using ANOVA.

## Data Availability

The data presented in this study are available upon request from the corresponding author. The data are not publicly available because of the protection of personal information.

## Appendix A

The study variables are as follows.

Satisfaction with the physical environment of local communities

In the 2023 KCHS utilized in this study, the social and physical environment includes the concepts of neighborhood trust, events (celebrations and condolences), and safety, the natural and living environment, public transportation, and medical services. In this study, the two factors related to neighbors (neighborhood trust and events) were excluded, as satisfaction with these factors was not measured.

Satisfaction with the natural environment

The natural environment includes factors such as air and water quality. Respondents were asked to answer “yes” or “no” to the question, “Are you satisfied with the natural environment in your neighborhood?”.

Satisfaction with the living environment

The living environment includes factors such as electricity, water supply and sewage systems, garbage collection, and sports facilities. Respondents were asked to answer “yes” or “no” to the question, “Are you satisfied with the living environment in your neighborhood?”.

Satisfaction with public transportation

Public transportation includes buses, taxis, subways, and trains. Respondents were asked to answer “yes” or “no” to the question, “Are you satisfied with the public transportation system in your neighborhood?”.

Satisfaction with medical services

Medical services include health centers, hospitals, traditional Korean medical clinics, and pharmacies. Respondents were asked to answer “yes” or “no” to the question, “Are you satisfied with the medical services in your neighborhood?”.

Number of days of vigorous physical activity

Vigorous physical activity refers to high-intensity physical activity, including running (jogging), hiking, fast cycling, fast swimming, soccer, basketball, skipping rope, squash, singles tennis, and heavy lifting, whether as part of occupational activities or sports. In the KCHS, the respondents were asked to indicate the number of days they had engaged in vigorous physical activity for at least 10 min in the past week, based on the question, “During the past 7 days, how many days did you engage in vigorous physical activity for at least 10 min that made you feel very tired or short of breath?”.

Number of days of moderate intensity physical activity

Moderate physical activity includes slow swimming, doubles tennis, volleyball, badminton, table tennis, and light lifting, whether as part of occupational activities or sports. In the KCHS, respondents were asked to indicate the number of days they had engaged in moderate physical activity for at least 10 min during the past week, excluding walking, based on the question, “During the past 7 days, on how many days did you engage in moderate physical activity for at least 10 min (excluding walking) that made you feel slightly tired or short of breath?”

Number of days of walking

Walking practice includes walking for commuting, school, travel, and exercise. Respondents were asked to indicate the number of days they had walked for at least 10 min at a stretch during the past week, based on the question, “During the past 7 days, on how many days did you walk for at least 10 min at a stretch?”

Number of days of flexibility exercise

Flexibility exercises include stretching and calisthenics. Respondents were asked to indicate the number of days they had engaged in flexibility exercises such as stretching or calisthenics during the past week, based on the question, “During the past 7 days, on how many days did you engage in flexibility exercises such as stretching or calisthenics?”. The response options were structured as follows: ① none, ② 1 day, ③ 2 days, ④ 3 days, ⑤ 4 days, and ⑥ 5 days or more. In the analysis process, the responses were coded as 0 for Bold text is required as a variable. I agree with simply removing the bold effect. “None”, 1 for “1 day”, 2 for “2 days”, 3 for “3 days”, 4 for “4 days”, and 5 for “5 days or more” to convert the variables into the number of days.

Self-rated health

SRH was assessed based on the question, “How would you rate your overall health?”. The response options were structured as follows: ① very good, ② good, ③ fair, ④ poor, and ⑤ very poor. In the analysis process, the responses were reverse-coded for ease of interpretation: 5 points for “Very good”, 4 points for “Good”, 3 points for “Fair”, 2 points for “Poor”, and 1 point for “Very poor”. This was carried out for ease of interpretation, so that a higher average score indicates a higher subjective health level for the group.

## References

[1] Kreiss D., McGregor S.C. (2024). A review and provocation: On polarization and platforms. New Media Soc..

[2] Burgess M.G., Van Boven L., Wagner G., Wong-Parodi G., Baker K., Boykoff M., Vandenbergh M.P. (2024). Supply, demand and polarization challenges facing US climate policies. Nat. Clim. Chang..

[3] Boxell L., Gentzkow M., Shapiro J.M. (2024). Cross-country trends in affective polarization. Rev. Econ. Stat..

[4] Collado Z.C. (2024). The Right to Healthcare Must Include the Right to Ease of Physical Access: Exploring Geography-Health Nexus in GIDA Communities in the Philippines. Int. J. Soc. Determ. Health Health Serv..

[5] Bijloo A. (2024). Residents’ Perspective on Their Utilisation of Community Centers in the Municipality of Zaltbommel, and How It Contributes to Their Personal Wellbeing. Master’s Thesis.

[6] Milton K., Cavill N., Chalkley A., Foster C., Gomersall S., Hagstromer M., Schipperijn J. (2021). Eight investments that work for physical activity. J. Phys. Act. Health.

[7] Bantham A., Ross S.E.T., Sebastião E., Hall G. (2021). Overcoming barriers to physical activity in underserved populations. Prog. Cardiovasc. Dis..

[8] Pulling Kuhn A., Cockerham A., O’reilly N., Bustad J., Miranda V., Loboda T.V., Hager E.R. (2021). Home and neighborhood physical activity location availability among African American adolescent girls living in low-income, urban communities: Associations with objectively measured physical activity. Int. J. Environ. Res. Public Health.

[9] Benyamini Y., Idler E.L. (1999). Community studies reporting association between self-rated health and mortality. J. Health Soc. Behav..

[10] DeSalvo K.B., Bloser N., Reynolds K., He J., Muntner P. (2006). Mortality prediction with a single general self-rated health question. J. Gen. Intern. Med..

[11] Maas J., Verheij R.A., Groenewegen P.P., De Vries S., Spreeuwenberg P. (2006). Green space, urbanity, and health: How strong is the relation?. J. Epidemiol. Community Health.

[12] Sugiyama T., Leslie E., Giles-Corti B., Owen N. (2008). Associations of neighbourhood greenness with physical and mental health: Do walking and social coherence mediate the relations?. J. Epidemiol. Community Health.

[13] Kim D., Kim S. (2007). The impact of urbanization and transportation development on health outcomes in Korea. J. Urban Health.

[14] Park S., Lee S. (2017). Public transportation and health outcomes in Korea: An analysis of the effects of public transportation satisfaction on subjective health. J. Transp. Health.

[15] Chang B.K. (2021). Differences in self-rated health and physical activity due to education level among Koreans: Understanding implications of physical education. Iran. J. Public Health.

[16] Bauman A., Phongsavan P., Lee I.M., Blair S., Manson J., Paffenbarger R.S. (2015). How can increase physical activity levels. Epidemiologic Methods in Physical Activity Studies.

[17] Korea Disease Control and Prevention Agency (2024). 2023 Korea Community Health Survey Guidelines for Using Raw Data.

[18] Cerin E., Nathan A., Van Cauwenberg J., Barnett D.W., Barnett A., Council on Environment and Physical Activity (CEPA)–Older Adults Working Group (2017). The neighbourhood physical environment and active travel in older adults: A systematic review and meta-analysis. Int. J. Behav. Nutr. Phys. Act..

[19] Sallis J.F., Cerin E., Conway T.L., Adams M.A., Frank L.D., Pratt M., Owen N. (2016). Physical activity in relation to urban environments in 14 cities worldwide: A cross-sectional study. Lancet.

[20] Sugiyama T., Cerin E., Owen N., Oyeyemi A.L., Conway T.L., Van Dyck D., Sallis J.F. (2014). Perceived neighbourhood environmental attributes associated with adults’ recreational walking: IPEN Adult study in 12 countries. Health Place.

[21] McNeill L.H., Kreuter M.W., Subramanian S.V. (2006). Social environment and physical activity: A review of concepts and evidence. Soc. Sci. Med..

[22] Tucker-Seeley R.D., Subramanian S.V., Li Y., Sorensen G. (2009). Neighborhood safety, socioeconomic status, and physical activity in older adults. Am. J. Prev. Med..

[23] Van Cauwenberg J., De Bourdeaudhuij I., De Meester F., Van Dyck D., Salmon J., Clarys P., Deforche B. (2011). Relationship between the physical environment and physical activity in older adults: A systematic review. Health Place.

[24] Foster C., Hillsdon M., Thorogood M., Kaur A., Wedatilake T. (2005). Interventions for promoting physical activity. Cochrane Database Syst. Rev..

[25] Bauman A.E., Reis R.S., Sallis J.F., Wells J.C., Loos R.J., Martin B.W. (2012). Correlates of physical activity: Why are some people physically active and others not?. Lancet.

[26] Giles-Corti B., Vernez-Moudon A., Reis R., Turrell G., Dannenberg A.L., Badland H., Owen N. (2016). City planning and population health: A global challenge. Lancet.

[27] Carlson J.A., Sallis J.F., Wagner N., Calfas K.J., Patrick K., Groesz L.M., Norman G.J. (2012). Brief physical activity-related psychosocial measures: Reliability and construct validity. J. Phys. Act. Health.

[28] Owen N., Humpel N., Leslie E., Bauman A., Sallis J.F. (2004). Understanding environmental influences on walking: Review and research agenda. Am. J. Prev. Med..

[29] Frank L.D., Andresen M.A., Schmid T.L. (2004). Obesity relationships with community design, physical activity, and time spent in cars. Am. J. Prev. Med..

[30] Feng X., Astell-Burt T. (2017). The relationship between neighbourhood safety and self-rated health: A multilevel analysis. J. Epidemiol. Community Health.

[31] Ross C.E., Mirowsky J. (2001). Neighborhood disadvantage, disorder, and health. J. Health Soc. Behav..

[32] Elo I.T., Mykyta L., Margolis R., Culhane J.F. (2009). Perceptions of neighborhood disorder: The association with self-rated health, and the mediating role of social ties. J. Urban Health.

[33] Stafford M., Chandola T., Marmot M. (2007). Association between fear of crime and mental health and physical functioning. Am. J. Public Health.

[34] Kim D. (2010). Neighborhood safety and its effect on self-rated health: A study of multilevel analysis. Soc. Sci. Med..

[35] Ulrich R.S. (1984). View through a window may influence recovery from surgery. Science.

[36] Mitchell R., Popham F. (2008). Effect of exposure to natural environment on health inequalities: An observational population study. Lancet.

[37] Kuo F.E., Sullivan W.C. (2001). Environment and crime in the inner city: Does vegetation reduce crime?. Environ. Behav..

[38] Kim D., Subramanian S.V., Kawachi I. (2006). Bonding versus bridging social capital and their associations with self-rated health: A multilevel analysis of 40 US communities. J. Epidemiol. Community Health.

[39] Kim S.J., Cho H. (2011). The effects of social support and neighborhood environments on mental health among older adults in Korea. J. Soc. Serv. Res..

[40] Sung H., Lee S. (2015). The influence of urban form on residents’ health outcomes: A focus on the Korean urban area. J. Urban Health.

[41] Park H., Kwon Y. (2017). Neighborhood socioeconomic deprivation and health: Exploring the relationship in the Korean context. Soc. Sci. Med..

[42] Cummins S., Stafford M., Macintyre S., Marmot M., Ellaway A. (2005). Neighbourhood environment and its association with self-rated health: Evidence from Scotland and England. J. Epidemiol. Community Health.

[43] Duncan D.T., Kawachi I., White K., Williams D.R. (2013). The geography of recreational open space: Influence of neighborhood racial composition and neighborhood poverty. J. Urban Health.

[44] Ellaway A., Macintyre S., Kearns A. (2001). Perceptions of place and health in socially contrasting neighbourhoods. Urban Stud..

[45] Wen M., Hawkley L.C., Cacioppo J.T. (2006). Objective and perceived neighborhood environment, individual SES and psychosocial factors, and self-rated health: An analysis of older adults in Cook County, Illinois. Soc. Sci. Med..

[46] Gong Y., Gallacher J., Palmer S., Fone D. (2014). Neighbourhood green space, physical function and participation in physical activities among elderly men: The Caerphilly Prospective Study. Int. J. Behav. Nutr. Phys. Act..

[47] Poor People’s Campaign (2018). The Souls of Poor Folk: Auditing America 50 Years After the Poor People’s Campaign Challenged Systemic Racism, Poverty, the War Economy/Militarism, Environmental Degradation & Our National Morality; Poor People’s Campaign. https://www.poorpeoplescampaign.org/resource/the-souls-of-poor-folk-audit/.

[48] Páez A., Mercado R.G., Farber S., Morency C., Roorda M. (2010). Relative accessibility deprivation indicators for urban settings: Definitions and application to food deserts in Montreal. Urban Stud..

[49] Li F., Harmer P.A., Cardinal B.J., Bosworth M., Johnson-Shelton D. (2009). Obesity and the built environment: Does the density of neighborhood fast-food outlets matter?. Am. J. Health Promot..

[50] Van Dyck D., Cardon G., Deforche B., De Bourdeaudhuij I. (2010). Neighborhood walkability and its particular importance for adults with a preference for passive transport. Health Place.

[51] Moudon A.V., Lee C., Cheadle A.D., Collier C.W., Johnson D., Schmid T.L., Weather R.D. (2005). Cycling and the built environment, a US perspective. Transp. Res. Part D Transp. Environ..

[52] Cerin E., Leslie E., du Toit L., Owen N., Frank L.D. (2007). Destinations that matter: Associations with walking for transport. Health Place.

[53] Lee J.H., Park H. (2015). Social networks, workplace environment, and subjective health among Korean adults. Soc. Sci. Med..

[54] Shi L., Starfield B. (2001). The effect of primary care physician supply and income inequality on mortality among black and white populations. Am. J. Public Health.

[55] Godlee F. (1996). Ensuring the quality of health care. BMJ.

[56] Matsumoto M., Inoue K., Takeuchi K. (2010). Rurality and physician supply in Japan: An analysis of the determinants of physician distribution. Health Policy.

[57] Flocke S.A., Stange K.C., Zyzanski S.J. (2000). The association of attributes of primary care with the delivery of clinical preventive services. Med. Care.

